# SynergyFinder 2.0: visual analytics of multi-drug combination synergies

**DOI:** 10.1093/nar/gkaa216

**Published:** 2020-04-04

**Authors:** Aleksandr Ianevski, Anil K Giri, Tero Aittokallio

**Affiliations:** Institute for Molecular Medicine Finland (FIMM), HiLIFE, University of Helsinki, FI-00290 Helsinki, Finland; Helsinki Institute for Information Technology (HIIT), Department of Computer Science, Aalto University, FI-02150 Espoo, Finland; Institute for Molecular Medicine Finland (FIMM), HiLIFE, University of Helsinki, FI-00290 Helsinki, Finland; Institute for Molecular Medicine Finland (FIMM), HiLIFE, University of Helsinki, FI-00290 Helsinki, Finland; Helsinki Institute for Information Technology (HIIT), Department of Computer Science, Aalto University, FI-02150 Espoo, Finland; Institute for Cancer Research, Department of Cancer Genetics, Oslo University Hospital, N-0310 Oslo, Norway; Oslo Centre for Biostatistics and Epidemiology (OCBE), Faculty of Medicine, University of Oslo, N-0317 Oslo, Norway

## Abstract

SynergyFinder (https://synergyfinder.fimm.fi) is a stand-alone web-application for interactive analysis and visualization of drug combination screening data. Since its first release in 2017, SynergyFinder has become a widely used web-tool both for the discovery of novel synergistic drug combinations in pre-clinical model systems (e.g. cell lines or primary patient-derived cells), and for better understanding of mechanisms of combination treatment efficacy or resistance. Here, we describe the latest version of SynergyFinder (release 2.0), which has extensively been upgraded through the addition of novel features supporting especially higher-order combination data analytics and exploratory visualization of multi-drug synergy patterns, along with automated outlier detection procedure, extended curve-fitting functionality and statistical analysis of replicate measurements. A number of additional improvements were also implemented based on the user requests, including new visualization and export options, updated user interface, as well as enhanced stability and performance of the web-tool. With these improvements, SynergyFinder 2.0 is expected to greatly extend its potential applications in various areas of multi-drug combinatorial screening and precision medicine.

## INTRODUCTION

Drug combinations have become a standard therapy for various complex diseases, including tuberculosis, malaria, HIV, and most of the advanced cancers (1–5). This is because multi-targeted treatments can lead to therapeutic benefits both by enhancing the treatment efficacy and by avoiding the acquisition of monotherapy resistance (6). Furthermore, in some cases, combinatorial treatments can be administered at lower doses of single drugs that would lead to intolerable dose ranges if used as monotherapies (7), thus reducing treatment side-effects. High-throughput combinatorial screening is an established approach to identify new synergistic drug combinations, i.e. combinations that result in a higher than expected effects (8). The degree of combination synergy, or antagonism, is quantified by comparing the observed drug combination response against the expected response, calculated using a reference model that assumes no interaction between drugs. The commonly-used reference models include the highest single agent (HSA) (9), Bliss (10), Loewe (11) and Zero interaction potency (ZIP) model (12). Drug combinations showing the highest synergy among all the combinations screened are then selected for further development and testing toward safe and effective treatment options. Hence, easy-to-use tools that enable unbiased identification of synergistic combinations from high-throughput experimental data are highly needed for systematic and reproducible discovery process.

To address this need, we implemented SynergyFinder (13), a web-application that enables researchers to pre-process, analyze and visualise pairwise drug combinations in an interactive manner. SynergyFinder scores drug combination synergy based on user's selection among the various reference models, and the web-tool supports interactive exploration and comparison of the synergy results. In addition to quantifying the overall synergies, SynergyFinder highlights the dose ranges with the strongest synergy or antagonism for more detailed analyses and interpretation about the clinical feasibility of the combination treatment. Since its initial release, SynergyFinder has been used in a wide range of precision medicine applications, including targeted drug combination discovery (14–16), drug resistance analysis (17), finding new vulnerabilities for mutated tumors (18,19) and comparative evaluation of synergy scoring models (20). Furthermore, the open-source SynergyFinder implementation has served as a building block and platform for the development of other drug combination analysis tools, including DECREASE (21) and SynToxProfiler (22).

Currently, the search for combinatorial discoveries is witnessing a paradigm shift from the traditional ‘two drugs in combination’ to the more complex ‘multi-drug cocktails’ (23–29). As a result, higher-order combination therapies involving three or more drugs have been approved or investigated for multiple diseases (e.g. cancers, HIV and tuberculosis) (1,3,5). For example, a so-called R-CHOP therapy that involves five drugs is an approved curative therapy for Diffuse Large B-Cell Lymphoma (30). The search for such higher-order synergistic combinations by industry and academia has also led to the generation of dose–response data for a large number of multi-drug combinations (31). However, most of the tools designed to analyse pairwise combinations do not naturally extend to scoring higher-order interaction, as the mathematical implementation of the reference models (e.g. HSA, Loewe and Bliss model) differs with the addition of each new drug. Furthermore, the visualization of higher-order interactions becomes increasingly complex, and non-intuitive visualizations can easily bias the conclusions about the degree of synergy. Additionally, understanding the contribution of each drug in a cocktail to the joint response requires a systematic assessment of all the sub-combinations (32). Therefore, there is a need for a software tool that cannot only assess synergy/antagonism of a large number of multi-drug combinations, but also enable an interactive exploration of the synergy patterns for an unbiased analysis of higher-order drug combination experiments.

Here, we present SynergyFinder version 2.0, an upgraded and improved web-application that enables the analysis of both pairwise and higher-order drug combination data. Based on the users’ requests, SynergyFinder 2.0 implements also novel and improved analysis and visualization options for multi-drug combination data, including automated outlier detection procedure, extended curve-fitting functionality, statistical assessment of replicate measurements, as well as many other enhancements appearing in the latest implementation of SynergyFinder.

## MATERIALS AND METHODS

### Overview of the extended functionality of SynergyFinder 2.0

Details of the original SynergyFinder implementation and its features for synergy assessment between two drugs have been described previously (13). Here, we primarily focus on the enhancements made to support synergy scoring for higher-order combinations, in addition to other web-tool improvements. More specifically, SynergyFinder 2.0 implements (i) efficient synergy estimation for multi-drug combinations, (ii) various curve-fitting algorithms for single drug dose–responses, (iii) automatic outlier detection in multi-drug combination screening data, (iv) novel visualization and export options and (v) statistical treatment of replicate measurements. A detailed comparison between the features of SynergyFinder release 1.0 and 2.0 is provided in Table 1.

**Table 1.** Comparison of specific features between SynergyFinder release 1.0 and release 2.0

Category	Release 1.0	Release 2.0
Synergy assessment	Two-drug combinations	Multi-drug combinations (two or more)
Outlier detection	No	Yes
Curve-fitting algorithm	Four-parameter logistic regression	Four-parameter logistic regression, linear regression, LOESS fitting
Replicate analysis	No	Yes
Minimum number of measured drug doses	Three doses	One dose
Visualization options	2D and 3D interactive surface plots, curve fit plots, heatmaps	2D and 3D interactive surface plots, curve fit plots, heatmaps, interactive 3D tensor plots, bar graphs
Reporting	Static and dynamic reports	Static, dynamic, and short reports. Simultaneous export of multiple synergy metrics and measured synergy values.

### SynergyFinder 2.0 analysis of multi-drug combinations

**Figure 1.**
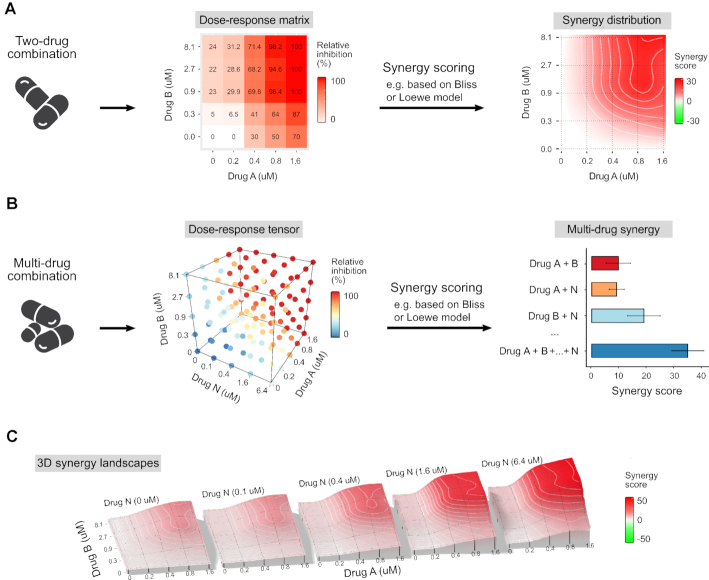
SynergyFinder 2.0 visual analytic options for (**A**) pairwise combinations and (**B**) higher-order combinations. (**C**) 3D synergy landscapes shown separately for each two-drug sub-combination.

Similar to SynergyFinder 1.0, version2.0 supports interactive analysis of two-drug combination data, based on the user-uploaded dose–response matrices (Figure 1A). As a result, interactive synergy distribution plots, together with summary synergy scores, are generated for each pair of drugs. In addition, SynergyFinder 2.0 supports the analysis of higher-order drug combinations by implementing interactive dose–response tensors for each triplet of the drugs (Figure 1B). Furthermore, barplots of synergy scores are produced separately for each sub-combination (pairs, triplets, etc.), depending on the number of drugs in the combinations. For more systematic analysis of the contribution of each drug to the joint higher-order combination effect, 3D synergy landscape plots for each of the two-drug sub-combinations are visualized enabling their further investigation (Figure 1C).

SynergyFinder 2.0. implements four reference synergy models (HSA, Bliss, Loewe and ZIP), and their extensions to calculate synergy scores for higher-order combination data. These models quantify the degree of synergy either as the excess over the maximum single drug response (HSA), multiplicative effect of single drugs as if they acted independently (Bliss), expected response corresponding to an additive effect as if the single drugs were the same compound (Loewe), and expected response corresponding to the effect as if the single drugs did not affect the potency of each other (ZIP). More specifically, the following higher-order formulations were used to quantify the drug combination synergy (*S*) for the measured multi-drug combination effect between *N* drugs ${E_{A,B, \ldots ,N}}$:

$$\begin{eqnarray*}\ {S_{HSA}} &=& {E_{A,B, \ldots ,N}}\ - \max ( {{E_A},{E_B}, \ldots ,{E_N}} ).\\\end{eqnarray*}$$

$$\begin{eqnarray*} {S_{BLISS}} &=& {E_{A,B, \ldots ,N}} - ( {E_A} + {E_B} + \ldots + {E_N} - {E_A}{E_b} \nonumber \\ &&- {E_A}{E_N} - {E_B}{E_N} - \ldots - {E_A}{E_B} \ldots {E_N} ).\\ \end{eqnarray*}$$

$$\begin{eqnarray*}\ {S_{LOEWE}} &=& \frac{a}{A}\ + \frac{b}{B} + \ldots + \frac{n}{N}.\\\end{eqnarray*}$$

$$\begin{eqnarray*} {S_{ZIP}} &=& {E_{A,B, \ldots ,N}} - \left( {\frac{{{{\left( {\frac{{{x_A}}}{{{m_A}}}} \right)}^{{{\rm{\lambda }}_A}}}}}{{1 + {{\left( {\frac{{{x_A}}}{{{m_A}}}} \right)}^{{{\rm{\lambda }}_A}}}}} + \frac{{{{\left( {\frac{{{x_B}}}{{{m_B}}}} \right)}^{{{\rm{\lambda }}_B}}}}}{{1 + {{\left( {\frac{{{x_B}}}{{{m_B}}}} \right)}^{{{\rm{\lambda }}_B}}}}} + \ldots} \right.\nonumber \\ && + \frac{{{{\left( {\frac{{{x_N}}}{{{m_N}}}} \right)}^{{{\rm{\lambda }}_N}}}}}{{1 + {{\left( {\frac{{{x_N}}}{{{m_N}}}} \right)}^{{{\rm{\lambda }}_N}}}}} - \frac{{{{\left( {\frac{{{x_A}}}{{{m_A}}}} \right)}^{{{\rm{\lambda }}_A}}}}}{{1 + {{\left( {\frac{{{x_A}}}{{{m_A}}}} \right)}^{{{\rm{\lambda }}_A}}}}}\frac{{{{\left( {\frac{{{x_B}}}{{{m_B}}}} \right)}^{{{\rm{\lambda }}_B}}}}}{{1 + {{\left( {\frac{{{{\rm{x}}_B}}}{{{m_B}}}} \right)}^{{{\rm{\lambda }}_B}}}}}\nonumber \\ && - \frac{{{{\left( {\frac{{{x_A}}}{{{m_A}}}} \right)}^{{{\rm{\lambda }}_A}}}}}{{1 + {{\left( {\frac{{{x_A}}}{{{m_A}}}} \right)}^{{{\rm{\lambda }}_A}}}}}\frac{{{{\left( {\frac{{{x_N}}}{{{m_N}}}} \right)}^{{{\rm{\lambda }}_N}}}}}{{1 + {{\left( {\frac{{{x_N}}}{{{m_N}}}} \right)}^{{{\rm{\lambda }}_N}}}}} \nonumber \\ && - \frac{{{{\left( {\frac{{{x_B}}}{{{m_B}}}} \right)}^{{{\rm{\lambda }}_B}}}}}{{1 + {{\left( {\frac{{{x_B}}}{{{m_B}}}} \right)}^{{{\rm{\lambda }}_B}}}}}\frac{{{{\left( {\frac{{{x_N}}}{{{m_N}}}} \right)}^{{{\rm{\lambda }}_N}}}}}{{1 + {{\left( {\frac{{{x_N}}}{{{m_N}}}} \right)}^{{{\rm{\lambda }}_N}}}}} - \ldots \nonumber \\ && \left.- \frac{{{{\left( {\frac{{{x_A}}}{{{m_A}}}} \right)}^{{{\rm{\lambda }}_A}}}}}{{1 + {{\left( {\frac{{{x_A}}}{{{m_A}}}} \right)}^{{{\rm{\lambda }}_A}}}}}\frac{{{{\left( {\frac{{{x_B}}}{{{m_B}}}} \right)}^{{{\rm{\lambda }}_B}}}}}{{1 + {{\left( {\frac{{{x_B}}}{{{m_B}}}} \right)}^{{{\rm{\lambda }}_B}}}}} \ldots \frac{{{{\left( {\frac{{{x_N}}}{{{m_N}}}} \right)}^{{{\rm{\lambda }}_N}}}}}{{1 + {{\left( {\frac{{{x_N}}}{{{m_N}}}} \right)}^{{{\rm{\lambda }}_N}}}}} \right). \end{eqnarray*}$$

Here, ${E_A},{E_B}, \ldots ,{E_N}$ are the measured responses of the single drugs, while *a*, *b* and *n* are the doses of the single drugs required to produce the combination effect ${E_{A,B, \ldots ,N}}$. For the ZIP model, ${x_N}$ is the dose of *N*^th^ drug fitted with four-parameter log-logistic (4PL) function, whereas ${m_N}$ is the dose that produces the half-maximum effect (also known as relative $E{C_{50}}$ or $I{C_{50}}$, depending on the readout), and ${{\rm{\lambda }}_N}$ is the shape parameter indicating the slope of the dose–response curve.

### Curve fitting and outlier detection in drug screening data

Accurate fitting of the dose–response curves is the first necessary step for any synergy assessment, since the fitted dose-response values are used for outlier detection and calculation of expected effects using the reference models (e.g. Bliss). The most commonly-used curve-fitting model for single-drug dose–responses is the four-parameter logistic (4PL) equation (33–35), which is also the default option in SynergyFinder. However, since some drug dose–responses may not accurately follow the 4PL model (e.g. U-shaped curves), SynergyFinder 2.0 allows users also to apply LOESS fit and linear regression as alternative algorithms for curve fitting. In case of replicate measurements, the dose–response curves are fitted using all the replicates, hence improving the robustness against outliers.

For automated detection of outlier measurements both in the combination and individual agent dose–response measurements, we utilized our recently-developed machine learning model, which is built on novel composite non-negative matrix factorization (cNMF) algorithm (21). More specifically, SynergyFinder 2.0 uses the cNMF algorithm to capture the overall combination patterns and to predict the full dose–response tensors for each combination. Then, the predicted responses are compared against the observed ones, and the user is alerted about any measurements that deviate >20% inhibition from the measured inhibition level as possible outlier measurements. The synergy calculations and visualizations can be performed with or without using the outlier measurements.

### SynergyFinder 2.0 input and output options

SynergyFinder 2.0 allows two possible drug screening data input file formats (Table and Matrix), with the file extensions either as *.xlsx, *.csv or *.txt files. More information about the input data format is given in the technical documentation available at https://synergyfinder.fimm.fi, ‘User guide’ button. Due to the various combination matrix layouts and experimental designs applied in screening projects, SynergyFinder 2.0 does not impose any restrictions on the drug combination design. Unlike the previous versions, the new version accepts both the ‘full combination designs’, where each drug is measured at multiple doses (36,37), as well as ‘partial combination designs’, where only a fixed single dose is used for any given drug (29,38). However, in the partial designs, only the Bliss and HSA synergy scores can be calculated, since Loewe and ZIP models require multiple doses for fitting dose–response curves of each drug in the combination. In the case of replicate measurements, SynergyFinder 2.0 also reports standard deviations for each synergy score, which enable statistical analyses of the combination effects.

For each multi-drug combination, SynergyFinder 2.0 quantifies the selected synergy scores for each combination of single-drug concentration mixtures, in addition to calculating the summary synergy level for the combination effect, i.e. the average of synergy scores over all the measured (non-outlier) concentrations. SynergyFinder 2.0 generates three types of summary PDF reports, which show subsets of the drug combinations, depending on the user's choices. For higher-order combinations, each triplet of drugs is visualized using 3D the dose–response tensor (Figure 1B), while separate 2D and 3D synergy landscapes between each pairs of two drugs are generated at different concentrations of *N*th drug (Figure 1C). The summary synergy scores between all the sub-combinations of drugs (pairs, triplets, etc) are visualized as summary barplots. Based on the user requests, one can also simultaneously export alternative summary tables (e.g. tables of multiple synergy scores and raw synergy results). These tables allow users to process the synergy results in other analytical or graphical software.

## CONCLUSIONS

We have implemented SynergyFinder version 2.0, a web-based application that enables the users to interactively assess, explore and visualize synergy in multi-drug combination assays. By allowing users to select various functions to fit the dose–response curves, cleaned by automated outlier detection procedure, SynergyFinder provides a flexible and robust solutions for an efficient and reproducible synergy scoring and visualization of multi-drug combinations from high-throughput screens. The use of multiple reference models to estimate synergy will also enable an unbiased evaluation of the pre-clinical significance of combinations toward further development for clinical applications. The web-tool facilitates both the drug combination discovery and screening programs, as it serves as the single point solution supporting multiple aspects of high-throughput combinatorial screening (e.g. outlier detection, curve fitting, and synergy scoring), thereby significantly reducing the time needed for the data analysis and interpretation. SynergyFinder 2.0 helps to assess the synergy scores for any *N*th order combinations, with the only restriction that all the corresponding single-drug responses should be measured at least with one concentration. We recommend using multi-dose assays for more accurate synergy landscape analyses, whereas fixed single-dose designs can be used for initial candidate screening. For two-drug combinations, one can also use our DECREASE model to predict the full dose–response matrices based on the more cost-effective fixed-dose or diagonal designs (21). We encourage users of SynergyFinder to continue leaving comments or suggestions for further improvements using the feedback form available on the website, as well as implement or request extended functionality through GitHub repository, with the aim of making SynergyFinder even more interactive and user-friendly.

## DATA AVAILABILITY

SynergyFinder 2.0 is an open-source software freely available at https://synergyfinder.fimm.fi without any login requirements. The software comes with example drug combination data, video tutorial and technical user instructions. The source codes of the web-application implementation are available at https://github.com/IanevskiAleksandr/SynergyFinder (under the BSD 3-clause license) to allow extension of the tool for further applications and integration with other software solutions.
