# Correction to ‘SynergyFinder 2.0: visual analytics of multi-drug combination synergies’

**DOI:** 10.1093/nar/gkac552

**Published:** 2022-06-17

*Nucleic Acids Research*, Volume 48, Issue W1, 02 July 2020, Pages W488–W493, https://doi.org/10.1093/nar/gkaa216

In the originally published version of this manuscript, there is an error in the Bliss and ZIP equations. There should be a ‘+’ sign instead of ‘-’, before the last term. However, the authors advise that the formulas are correct in the codes used for calculation on the website, so the error doesn’t affect any previous or future analysis with SynergyFinder.

These details have been corrected only in this correction notice to preserve the published version of record.

